# Nausea and vomiting of pregnancy and resource implications: the NVP Impact Study

**DOI:** 10.3399/bjgp18X700745

**Published:** 2018-12-18

**Authors:** Roger Gadsby, Verity Rawson, Edward Dziadulewicz, Ben Rousseau, Hannah Collings

**Affiliations:** Warwick Medical School, University of Warwick, Coventry.; Alliance Pharma Ltd, Chippenham, Wiltshire.; Alliance Pharma Ltd, Chippenham, Wiltshire.; Adelphi Values PROVE, Bollington, Cheshire.; Adelphi Values PROVE, Bollington, Cheshire.

**Keywords:** general practice, nausea, pregnancy, primary health care, vomiting

## Abstract

**Background:**

Nausea and vomiting of pregnancy (NVP) is the most prevalent medical condition associated with pregnancy. The Royal College of Obstetricians and Gynaecologists published its first guidelines for management of NVP in 2016, although many current treatments are off label, with only one recently licensed treatment for NVP in the UK.

**Aim:**

To identify the current practices for NVP management across the patient pathway, and estimate the economic burden to NHS services.

**Design and setting:**

This was an observational, retrospective research study conducted in the Newcastle Gateshead Clinical Commissioning Group (CCG) health economy area in England.

**Method:**

Data were collected from GP practices, local hospital datasets, ambulance services (April 2013–March 2016), and the Hospital Episode Statistics dataset (2006–2016).

**Results:**

Eight GP practices participated in the study. In all, 15.2% of the total pregnant population presented with NVP. Treatment varied significantly between GP practices, and 33.6% of women re-presented to their GP. There was an annual increase in women admitted to hospital for NVP symptoms, with increasing length of stay per admission. Almost half (44.6%) of the calls to 999/111 from women experiencing NVP symptoms resulted in an ambulance dispatch. The annual cost of NVP to this health economy was estimated to be £199 804, which crudely extrapolates to £25 758 731 at UK level. Due to underestimations of costs, the impact to the UK NHS could be up to £62 373 961.

**Conclusion:**

There is considerable variation in current management practices for NVP outside of recently published guidelines, and this may result in substantial resource use and avoidable financial impact to the NHS.

## INTRODUCTION

Nausea and vomiting of pregnancy (NVP) is the most prevalent medical condition associated with pregnancy, affecting up to 80% of pregnant women.^1^ The aetiology of NVP is unknown, and symptoms range in severity from mild nausea to the most severe form of vomiting, known as hyperemesis gravidarum (HG). This occurs in 0.3–3.6% of pregnancies, and can potentially result in metabolic disturbances, including dehydration and ketosis.^2^^,^^3^ NVP can also impact quality of life, reducing ability to carry out daily parenting or work tasks, with increased reliance on childcare arrangements.^4^ Up to 35% of women with NVP report depression.^5^

The Royal College of Obstetricians and Gynaecologists (RCOG) published its first guidelines for the management of NVP in 2016.^2^ There is currently only one licensed treatment for NVP in the UK,^6^ with many treatments used off-licence. The RCOG recommends a range of interventions beginning with conservative management. If medication is warranted, antiemetics such as histamine H1 receptor antagonists and phenothiazines are recommended first-line treatments; they can be used in combination for women who do not respond to monotherapy. The guidelines also suggest second-line treatments, with corticosteroids only to be used as third-line where standard therapies fail.^2^

Although there has been research into NVP treatment and its impact in pregnant women, there is limited understanding of the impact on healthcare resources and overall economic burden associated with NVP management.^5^ A study by Gadsby *et al* included a literature review of factors influencing the clinical presentation of NVP.^7^ The authors estimated the cost to the NHS for NVP hospital admissions in 2003–2004 to be £36 481 745.^7^

Based on the high prevalence of the condition, lack of licensed pharmacotherapy options, non-specific guidance, and paucity of data on current management and costs, this study of a large UK population sample was conducted to understand NVP management and to assess the impact on NHS services and associated costs.

## METHOD

This study focused on the health economy within one NHS clinical commissioning group (CCG) in England. The Newcastle Gateshead CCG health economy was selected as it provides a wide range of patient demographics likely to be representative of the national population.

Data included in the study were 999/111 services, primary care data (GP practice data), and secondary care data (NVP hospital admission data and informal interviews with a midwife employed by the hospital trust). An independent healthcare consultant assisted in collection of data from these sectors, using the methodologies explained further on. In accordance with NHS information governance regulations, no patient-identifiable information was obtained.

**How this fits in**

Although nausea and vomiting of pregnancy (NVP) is the most prevalent medical condition and most common indication for hospital admission during pregnancy, little is known about its economic burden and how healthcare resources are utilised. This study provides insights into both of these aspects of NVP, and its current management in the UK.

For primary care data collection, eight GP practices representative of different socioeconomic and ethnic backgrounds were invited to participate. These GP practices were selected by an expert researcher with knowledge of the local primary care services in order to provide a representative study sample covering a broad range of population demographics. Data were collected for the selected study period (1 April 2014 to 31 March 2015). This included the practice population (at 31 March 2015), total number of women who were pregnant at any point during the data collection period, and details for individual women presenting with NVP. Data recorded for each patient included age, ethnicity, week of pregnancy, gravidity, parity, number of consultations for NVP during the pregnancy and with which healthcare professional (GP, nurse, or midwife), and available notes from the consultation.

Data were extracted from the IT systems at the practice, and free-text boxes from each of the patient notes were manually assessed to extract relevant information. Secondary care data were collected from two sources: an acute secondary care hospital (one of the two within the Newcastle Gateshead CCG) and the Hospital Episode Statistics (HES) dataset.

Data from the hospital for 3 financial years (1 April 2013–31 March 2016) were included in the analysis, covering all NVP admissions within the five International Classification of Diseases 10^th^ Revision (ICD-10) codes for ‘excessive vomiting in pregnancy’: O21.0 — mild hyperemesis gravidarum; O21.1 — hyperemesis gravidarum with metabolic disturbance; O21.2 — late vomiting of pregnancy; O21.8 — other vomiting complicating pregnancy, and O21.9 — vomiting of pregnancy, unspecified.

Data from the HES database relating to admissions for the five NVP ICD-10 codes were analysed for all 65 GP practices in the health economy between 2006 and 2016. These data reported the number of admissions and the length of stay for all admissions. Due to NHS Digital information governance and data-handling regulations, complete granularity of the data originally requested for the locality could not be supplied (where activity values were <5), so totals are presented as a minimum of the precise total.

Anonymised data from the CCG ambulance service were collected with permission from the North East Ambulance Service NHS Foundation Trust (NEAS) records. This service provides the 999 emergency and 111 non-emergency telephone service. Data were provided from 1 April 2014–31 March 2015.

All data were collated and analysed in Microsoft Excel. Estimated unit costs for services across primary care, secondary care, and ambulance services used a recently published local health technology costings of NVP by O’Donnell *et al* 4 and the Personal Social Services Research Unit (PSSRU) annual listing from 2016.^8^ Full details of costs used are presented in Appendix 1.

## RESULTS

### Primary care management of NVP

All eight GP practices accepted the invitation to participate, covering a registered patient population of 59 591 (11.7% of the total CCG population). Patient demographics and GP practice profiles are available from the authors on request, and included a variety of socioeconomic and ethnic backgrounds. Within the 1-year study period, this population included 804 pregnant women, 122 (15.2%) presenting with NVP.

Most women presenting with NVP had a GP consultation (55.0%), with the remainder presenting to a midwife (14.0%), or with more than one healthcare professional present (including a GP [30.0%]). The healthcare professional was not recorded in the case of two women (1.0%).

The 122 women were involved in a total of 194 primary care presentations for NVP, with 100 treatment descriptions recorded in the free-text field of the clinician notes (Table 1); 51 (51.0%) of the treatment descriptions were conservative management measures, 22 (22.0%) first-line pharmacotherapies, and four (4.0%) were second-line pharmacotherapies. No third-line pharmacotherapy was issued.^5^ There were also 17 (17.0%) other medicines issued that do not feature in the 2016 RCOG guidelines, four (4.0%) recommendations for hospital referral, and two (2.0%) where additional diagnoses were considered (Table 1). Treatment recommendations were not uniformly prescribed across all of the practices of the study, with clustering of similar treatment regimens that varied from one GP practice to another (Table 2). There was also a range of frequencies by which women re-presented to their GP surgery (range = 1–7), with 33.6% of women having visited their GP on more than one occasion for NVP in the same pregnancy.

**Table 1.** Frequency of prescribed treatment regimens from the eight GP practices in the primary care sample

**Treatment regimen (according to RCOG guidelines)**	**Treatment**	**Frequency**	**Total treatment regimens, %**
**Conservative measures**	Advice mentioned	6	51.0
	‘Assess’, ‘review’, ‘f2f’, or ‘consult’	6	
	Berocca^®^	1	
	Diet advice (including ‘small amounts’)	8	
	Fluids advised	10	
	Ginger	5	
	Leaflet	3	
	Monitored	7	
	Rest	1	
	No treatment	4	

**First-line pharmacotherapy**	Cyclizine	8	22.0
	Prochlorperazine	11	
	Promethazine	3	

**Second-line pharmacotherapy**	Domperidone	1	4.0
	Metoclopramide	2	
	Ondansetron	1	

**Third-line pharmacotherapy**	–	0	0

**Other medicines prescribed^a^**	Antibiotics (including amoxicillin and cefalexin)	3	17.0
	Buscopan	1	
	Codeine	1	
	Folic acid	4	
	Ranitidine	2	
	Sertraline	1	
	Vitamin D	4	
	Paracetamol	1	

**Hospital referred (including A&E)**	**–**	4	4.0

**Additional diagnosis considered**		2	2.0

**Total**		**100**	

a Treatment not mentioned in the RCOG guidelines, prescribed according to GP’s own judgement. A&E = accident and emergency. f2f = face-to-face consultation. RCOG = Royal College of Obstetricians and Gynaecologists.

**Table 2.** Frequency of recorded treatments given or actions taken from each of the eight GP practices in the Newcastle Gateshead CCG

**Treatment or action taken**	**GP practices**							
	**A**	**B**	**C**	**D**	**E**	**F**	**G**	**H**
**Additional diagnosis considered**	1			1				
**Advice given**				2				4
**Antibiotics (including amoxicillin and cefalexin)**				3				
**‘Assess’, ‘review’, ‘f2f’, or ‘consult’**	2			4				
**Berocca^®^**				1				
**Buscopan**						1		
**Codeine**		1						
**Cyclizine**	1		1	4		2		
**Diet advice (including ‘small amounts’)**	4		1	3				
**Domperidone**						1		
**Fluids advised**	7			3				
**Folic acid**				4				
**Ginger**			3	2				
**Hospital referral**	1			2		1		
**Leaflet**				2		1		
**Metoclopramide**						1		1
**Monitored**		7						
**No treatment**	4							
**Ondansetron**				1				
**Paracetamol**				1				
**Prochlorperazine**	2	1	3			5		
**Promethazine**		2				1		
**Ranitidine**				1		1		
**Rest**	1							
**Sertraline**		1						
**Vitamin D**				4				

*CCG* = *clinical commissioning group. f2f* = *face-to-face consultation.*

### Secondary care management of NVP

#### Hospital

In the hospital setting, the total number of NVP episodes increased over the 3 years of the study period, with an 89.5% increase between 2013–2014 and 2014–2015, and a 13.9% increase between 2014–2015 and 2015–2016. There was also a year-on-year increase in the number of patients admitted for NVP symptoms over the study period. The average length of stay per hospital admission increased slightly across the 3 study years (1.16, 1.32, and 1.52 days in 2013–2014, 2014–2015, and 2015–2016, respectively) with a mean length of stay of 1.33 days.

#### HES dataset

There were 509 214 patients registered in Newcastle Gateshead CCG. The annual number of births for the CCG health economy increased and the number of admissions for NVP fluctuated over the 10-year study period, increasing in recent years. The average length of hospital stay in the CCG was consistent over time, with 1.4 days per admission in 2013–2014, 1.3 days per admission in 2014–2015, and 1.5 days per admission in 2015–2016, with a mean length of stay of 1.36 days.

### Ambulance services for NVP

In the 12-month period studied, there were 145 999 emergency calls and 198 111 non-emergency calls made to the NEAS for pregnant women experiencing NVP symptoms. In total, 44.6% of these calls resulted in an ambulance being dispatched; 80.7% of the 999 calls led to an ambulance visit and 18.2% of 111 calls, the combined data suggesting that 153 women with NVP received an ambulance during the 1-year study period.

### Economic burden of NVP

Estimated costs of NVP for the Newcastle Gateshead CCG health economy over the 1-year study period are presented in Table 3. Data from the primary care study sample were extrapolated to CCG level using a multiplier of 8.545 based on the difference in population (59 591 [GP cohort] to 509 214 [CCG]). This equates to 1658 consultations for NVP: 1409 to a GP, and 249 with a midwife. The total estimated cost of primary care services was £72 369.

**Table 3.** Estimated costs of NVP for the Newcastle Gateshead CCG health economy over the 1-year study period

**Service**	**Item**	**Number**	**Costs per unit, £**	**Total cost, £**
**Primary care**	GP appointment	1409	45	63 405
	Midwife consultation	249	36	8964
**Secondary care**	Inpatient admissions	93	850	79 050
	A&E attendances	33	290	9570
**Ambulance service**	999 call	145	7	1015
	111 call	198	7	1386
	Ambulance response	153	238	36 414
**Total, £**				**199 804**

*A&E* = *accident and emergency. CCG* = *clinical commissioning group. NVP* = *nausea and vomiting of pregnancy.*

Following a GP consultation, two women in the study sample were directed to attend accident and emergency (A&E). This extrapolated to 17 women (using the multiplier of 8.545 as outlined above) at CCG level. Following a 999/111 call to ambulance services within the CCG as a whole, a further 16 women were directed to attend A&E. Therefore there was a total of 33 assumed presentations to A&E at a CCG level. The total estimated cost of A&E presentations at CCG level was £9570.

Data from HES reported a minimum of 93 inpatient admissions in the CCG population with an NVP primary diagnosis. The total estimated cost of secondary care services was £88 620. The combined total of 999 and 111 calls relating to NVP in the CCG was 343, resulting in 153 ambulance dispatches. The estimated total cost of ambulance services was £38 815.

Based on this, the estimated financial resource cost to Newcastle Gateshead CCG health economy was £199 804 over a 1-year period. This gives an estimated total cost of NVP in England of £21 686 726 (based on the CCG population of 509 214, England population of 55 268 100, multiplier of 108.54 [2 decimal places {dp}]),^9^ and £25 758 731 for the UK (based on UK population of 65 648 100, multiplier of 128.92 [2dp]).^10^

Hospital admission rates in this CCG health economy are five times lower than the highest ranked CCG, and were 45% of the total minimum financial burden of NVP in this area. Using this proportion, the minimum financial burden of NVP in the UK may be as high as £62 373 961.

## DISCUSSION

### Summary

The results of this observational study demonstrate a substantial variation in the management of NVP. The primary care analysis indicated that an average of 15.2% of pregnant women presented with NVP, which is comparable to the 20% reported in the literature.^11^ With the RCOG guidelines published only a few months before the study was conducted, variation in the management of NVP in the primary care setting may be reflective of the absence of guidelines for the management of NVP at the time of the study, and it may have improved since.^2^ This may also account for the clustering of treatment descriptions recorded across primary care practices, where GPs were more likely to prescribe treatments they are familiar with in the absence of guidelines. It is also noted that use of an objective measure of NVP severity is recommended by RCOG guidelines as a method of monitoring treatment. However, this study did not identify any recorded NVP severity from the consultation notes, either as ‘severe’ or ‘moderate’, or via a validated assessment tool such as the Pregnancy-Unique Quantification of Emesis/Nausea Index (PUQE score). There was a high frequency of repeat visits to GP practices, with some women visiting up to seven times in the same pregnancy. This may be a result of inadequate or unsuccessful management of NVP in primary care, with women visiting repeatedly with unresolved symptoms.

The secondary care dataset showed consistency in the mean length of stay (1.36 days) for patients admitted with NVP. The HES data for the CCG reported 5326 births in 2014–2015, while a national data comparison estimates 6870. Although these figures are similar, the difference may be due to women who were pregnant in 2014–2015 actually giving birth in 2015–2016, women possibly moving CCG prior to birth, or women leaving practice lists at the time of data collection.

The midwife interviewed at the hospital discussed how symptom reporting may be used to seek additional support lacking in the daily lives of these women, corresponding to the feelings of helplessness and isolation reported in the literature.^12^ The midwife also affirmed that early pregnancy assessment units should be the first point of contact for pregnant women.

Although only 145 of the >45 000 monthly 999 calls made to the NEAS ambulance services were related to NVP, 80.7% of NVP calls resulted in an emergency ambulance call dispatch. Similarly, 198 of the 50 000 monthly 111 calls were related to NVP, with 18.2% of these calls resulting in an emergency ambulance dispatch. Therefore, although there is a relatively small total number of calls, a large proportion resulted in an ambulance dispatch. The combined data from the 999 and 111 calls suggested that 153 women with NVP received an ambulance dispatch during the 1-year study period, which relates to one ambulance almost every other day in this CCG. This demonstrates the substantial resource use that could potentially be avoided by earlier or more guideline-driven interventions.

### Strengths and limitations

This is the first study that has looked at the impact of NVP across an NHS primary, community, and secondary care sector in the UK. It is the first study to look at the economic costs of NVP across all NHS sectors.

The patient demographics of the study cohort represented a wide range of the ethnic and socioeconomic backgrounds found within the national population of the UK, providing a level of reliability to the data estimates. For the primary care population, this study contained an 11.7% sample of the whole CCG population, and practices were selected based on the range of patient demographics and variance in practice size, to reflect the socioeconomic mix of the wider CCG population. These results, therefore, provide a basis for estimating the national burden of NVP. However, it is recognised that these real-world data are not a statistically representative sample, and this could be a potential limitation of the study. Other limitations include the underestimation of the accurate totals from the HES dataset due to data-handling regulations of low patient numbers, the exclusion of the additional healthcare professionals present at primary care consultations for approximately 30% of women, exclusion of all prescription costs, and the fact that a large proportion of treatment outcomes from two practices (Practices E and G) were unrecorded. Extrapolating the total cost for the CCG health economy to the national population likely underestimates the real total. Hospital admission rates in this CCG health economy are one of the lowest per head of population in England and Wales, ranked 162 out of 209 CCGs, which is five times lower than the highest ranked CCG. Therefore, these figures are a likely underestimate of the true national average. The authors’ analysis found that hospital admissions accounted for 45% of the total minimum financial burden of NVP for the CCG health economy. Using this proportion, it is therefore estimated that the minimum financial burden of NVP in the UK may be as much as £62 373 961.

The study period differed between sources due to limitations in the ability to gather complete individual data sets. Although the data included in this analysis were collated from the CCG population, the primary care data were taken from a representative sample due to time and resource constraints; thus, unavoidable limitations exist when extrapolating these data to CCG levels.

### Comparison with existing literature

This is the only study of its kind in this therapeutic area that has estimated the costs of the condition across primary and secondary care sectors of the NHS. Integral to the results of this study, the authors have used the information calculated by O’Donnell *et al*,^4^ which developed a portfolio of healthcare financial costs for women with NVP in the Newcastle Gateshead region.

### Implications for research and practice

Recommendations for further research include gaining a greater understanding of the variation in coding and management of NVP in primary care by assessing coding variations in other health economies to gain a more accurate picture of the true burden of NVP, reviewing primary care templates to understand the advice provided, building authoritative primary care guidelines for NVP management to reduce variability in treatment, and ensuring consistency in the care received by women with these symptoms. Further research into the economic burden of NVP may be directed towards accurately determining the variance in NVP hospital admission costs at a patient level, including factors such as severity of symptoms, length of stay, and use of outpatient services. It would also be of interest to understand the impact of NVP management on outcomes for the individual patient, especially in terms of repeat presentations. The costs of NVP for the NHS are considerable, and any effective improvements in treatment and management will benefit sufferers, and potentially reduce the economic burden of the condition.

## Appendix 1. Healthcare resources unit costs in 2016^4^^,^^7^

**Table:**

**Service**	**Item**	**Notes**	**Cost, £**
**Primary care**	GP appointment	Per consultation lasting 11.7 minutes	45^a^
	Community midwife	Per hour	36^b^
**Secondary care**	Inpatient admission	Short stay 2-night inpatient admission, including IV rehydration, IV/oral antiemetics, appropriate blood and urine tests, an ultrasound scan, thromboprophylaxis, and thiamine supplementation	Up to 850^a^
	A&E attendance^c^	Including IV rehydration, IV antiemetics, appropriate blood and urine tests, and ultrasound scans	**290^a^**
**Ambulance service**	999 call	–	7^b^
	111 call	–	**7^b^**
	Ambulance — see & treat & convey	–	238^b^

a 2016 costs specific to NVP management in Newcastle and Gateshead region.^4^

b 2016 costs from the PSSRU.^7^

c *Estimated A&E attendance costing from known outpatient costing; likely an underestimate. A&E* = *accident and emergency. IV* = *intravenous. NVP* = *nausea and vomiting of pregnancy. PSSRU* = *Personal Social Services Research Unit.*
